# *Helicobacter pylori* treatment: antibiotics or probiotics

**DOI:** 10.1007/s00253-017-8535-7

**Published:** 2017-10-26

**Authors:** Kamila Goderska, Sonia Agudo Pena, Teresa Alarcon

**Affiliations:** 1 Faculty of Food Science and Nutrition, Institute of Food Technology of Plant Origin, Department of Fermentation and Biosynthesis, Poznan University of Life Sciences, Wojska Polskiego 31, 60-624 Poznan, Poland; 2 Department of Microbiology, Hospital Universitario de La Princesa, 28006 Madrid, Spain

**Keywords:** *Helicobacter*, Antibiotics, Probiotics, Intestinal microbiology, Antimicrobials

## Abstract

Treatment of *Helicobacter pylori* infection is important for the management of gastrointestinal disorders such as peptic ulcer and gastric cancer. Due to the increase in the prevalence of *H. pylori* resistance to antibiotics, triple therapy with clarithromycin is no longer the best treatment for H*. pylori*, especially in some areas where the local resistance to this antibiotic is higher than 20%. Alternative treatments have been proposed for the eradication of *H. pylori*. Some of them including novel antibiotics or classical ones in different combinations; these treatments are being used in the regular clinical practice as novel and more effective treatments. Others therapies are using probiotics associated to antibiotics to treat this infection.

The present article is a revision of *H. pylori* eradication treatment, focusing on emerging approaches to avoid the treatment failure, using new therapies with antimicrobials or with probiotics.

## Introduction

*Helicobacter pylori* is a common bacteria infecting about half of world’s population, with higher prevalence in developing countries, where *H. pylori* could infect up to 80% of the population (Moayyedi and Hunt 2004), than in developed ones.

*H. pylori* is associated with the development of gastrointestinal disorders as chronic gastritis, peptic ulcer, and gastric adenocarcinoma (Kuipers 1997). *H. pylori* is also involved in the development of other extra-gastric disorders such as mucosa-associated lymphoid tissue lymphoma (MALT), idiopathic thrombocytopenic purpura, vitamin B_12_ deficiency, and iron deficiency (Kuipers 1997). Eradication of *H. pylori* could help in the management of these *H. pylori*-associated disorders.

For the last two decades, the recommended treatment for *H. pylori* eradication is the standard triple therapy (Papastergiou et al. 2014a, b), using a proton pump inhibitor or ranitidine bismuth citrate, combined with clarithromycin and amoxicillin or metronidazole.

During the 90’s, due to the fact that these treatments reached high eradication rates [more than 90%] together with the safety profile, these triple therapies had a very high acceptance among clinicians (Malfertheiner et al. 2007). The efficacy of these triple regimens has decreased lately to rates lower than 70%, due to *H. pylori* resistance to key antibiotics, mainly clarithromycin, but also metronidazole and levofloxacin (Agudo et al. 2010a, b; De Francesco et al. 2009).

These low rates of successful treatment are not acceptable under the Maastricht consensus which points out that rates consistently below 80% by intention-to-treat are not acceptable for treating *H. pylori* (Graham et al. 2007). Information about local resistant to antibiotics should be taken into account before establishing a treatment plan for the patient to avoid repeated treatments. Several expositions to antibiotic treatments could result in more side effects and a decrease in the percentage of antibiotic resistance.

For this reason, this review is an overview of *H. pylori* eradication focused on second-line therapies that are used such as sequential therapy and quadruple therapy. However, due to the increase of the antibiotic resistance, some studies have started to focus on probiotics, as a therapeutic approach. Probiotics are defined as living microbial species that can include anti-inflammatory and anti-oxidative mechanisms that may improve bowel microecology and general health (Lu et al. 2016). Probiotics are live microorganisms, which when administered in adequate amounts confer a health benefit on the host. The most used probiotic bacteria are *Lactobacillus* and *Bifidobacterium* (Ruggiero 2014). Probiotics could improve *H. pylori* eradication and reduce side effects during therapy (Kim et al. 2008). A part of this revision will be focused on using of probiotics against *H. pylori*.

### *H. pylori* resistance to antibiotics

#### Classical treatment

During the 90s, the standard triple therapy was the gold standard in the treatment of *H. pylori* infections. The standard triple therapies are based on a proton pump inhibitor, clarithromycin, and amoxicillin or metronidazole. The increase in the prevalence of resistance to these antibiotics, especially to the key antibiotic, clarithromycin, has decreased the efficacy of standard regimens (Malfertheiner et al. 2002).

In a recent systematic review, the global incidence of primary *H. pylori* resistance to clarithromycin has been reported to be as high as 17.2%, showing an increase worldwide (Kuipers 1997). The prevalence of *H. pylori* resistance to clarithromycin varies among different countries, such as 10.6 to 25% in North America, 16% in Japan, and 1.7 to 23.4% in Europe (Elitsur et al. 2006; Horiki et al. 2012; Koletzko et al. 2006). This disparity in resistance rates seems to be correlated to the national level of macrolide consumption and different policies for antibiotic consumption in different countries (Agudo et al. 2010a, b), for example, 49% of clarithromycin resistance has been reported in some Spanish areas, but only 1% in the Netherlands, reflecting a stricter Northern European policy for antibiotic use than in Southern European countries (Seck et al. 2013). New macrolides were marketed in Europe at the beginning of the 90’s; patients were exposed to macrolides in order to treat respiratory infections with antibiotics of this group. Additional aspects such as geographic features, virulence factors of *H. pylori* strains, or some host aspects [age, place of birth] could contribute to the significant variation in the prevalence of antibiotic resistance (Van Doorn et al. 2000).

Metronidazole is a key component included in the triple therapies (Malfertheiner et al. 2002) which is associated to a high level of resistance. The prevalence of metronidazole resistance has been estimated to be from 17 to 44% for Europe and America, respectively (Ogata et al. 2013; De Francesco et al. 2010). The highest level of resistance to this antibiotic in Europe has been reported in Western Europe, where 20 to 45% of the *H. pylori* isolates are metronidazole-resistant (Lopez-Brea et al. 2001). The percentage of metronidazole resistance in developing countries has been reported from 50 to 100%, instead of being up to 90% in Africa (Falsafi et al. 2004). The resistance to metronidazole is so high in developing countries because this antibiotic is widely used to treat parasitic and/or gynecological infections in female patients (John et al. 2006). Also, there are some studies describing that metronidazole resistance being linked to the virulence strain factors, the strains without *cagA* gene being more resistant (Taneike et al. 2009). The lower rates related to metronidazole resistance have been reported in Japan, around 10% (De Francesco et al. 2010).

Based on these publications, standard triple therapies may not be recommended anymore for empiric use. Due to the high level of resistance to the two key antibiotics of standard triple therapies, clarithromycin and metronidazole, and the different patterns of resistance in different populations, standard triple therapies should be adapted to the local resistance pattern, and when possible, treatment should be based on susceptibility data obtained by testing the strain after culture.

Alternative strategies are being implemented in clinical practice to treat *H. pylori*-resistant strains. This included development and use of novel and more effective treatments and use of probiotics to improve the eradication regimens and decrease the antibiotic side effects.

During this revision, new treatment against *H. pylori* and probiotics therapies will be discussed.

#### Bismuth quadruple therapy

This therapy contains two antibiotics, tetracycline and metronidazole, plus bismuth and PPI for 14 days (Harb et al. 2015). This therapy is preferred as a first-line treatment option for areas with a high incidence of clarithromycin resistance and also as second-line therapy when first treatment with the classical triple therapy against *H. pylori* was failed (Papastergiou et al. 2014a, b). This therapy works totally independent of clarithromycin, the most problematic antibiotic in terms of resistance. Related to metronidazole, the use of high doses and prolonged treatment duration allows minimizing the impact on metronidazole-resistant strains, providing high eradication rates even in areas with high level of resistance to this antibiotic (Lee et al. 2014).

The potential toxicity of bismuth as well as the non-availability of bismuth salts or tetracycline in some countries is the main impediment related to this therapy. In some studies, tetracycline was substituted by amoxicillin (Perri et al. 2002).

Nine randomized controlled trials were analyzed in a meta-analysis (Luther et al. 2010), comparing bismuth quadruple therapy and clarithromycin triple therapy; they found that bismuth quadruple therapy achieved eradication in 78.3% of patients, whereas clarithromycin triple therapy achieved an eradication rate of 77%. The conclusion was that quadruple and triple therapies have similar eradication rates as primary therapy for *H. pylori* infection. On the other hand, the efficacy of this therapy has been confirmed as second-line treatment in a meta-analysis based on 30 studies showing 77% of eradication after failure of standard triple therapy (Gisbert 2012).

#### Sequential therapy

Sequential therapy uses the same antibiotics as standard triple therapy, but they are given them sequentially: 5 days with a PPI plus amoxicillin, followed by 5 days of PPI plus clarithromycin and amoxicillin (Kuipers 1997).

Amoxicillin is administrated first, due to the fact that amoxicillin disrupts the bacterial cell walls to prevent the development of efflux channels transferring the rest of the antibiotics out of bacteria (Webber and Piddock 2003). There were some studies published, the majority of them in Italy, where the result of sequential therapy was superior to normal standard triple therapy. More recent data from studies developed in South America and Asia shows eradication rates lower than 80% (Kuipers 1997).

In a recent meta-analysis, 36 randomized clinical trials were reviewed. The eradication rate was 84.1% for the sequential therapy in comparison with 75.1% for the standard triple therapy. Sequential therapy seems to be more effective against patients with the single clarithromycin-resistant strain; eradication rates were 80.9% for sequential therapy and 40.7% for standard triple therapy (Feng et al. 2015).

#### Non-bismuth quadruple therapy

It is another valid therapy in countries with high incidence to clarithromycin resistance. This therapy contains PPI [but without bismuth], clarithromycin, amoxicillin, and metronidazole for 10 days. The main disadvantage of this treatment is the large number of pills in comparison with other therapies (Papastergiou et al. 2014a, b).

In a meta-analysis carried out in 2012 including randomized controlled studies comparing non-bismuth quadruple therapy and the standard triple therapy, the eradication rate was 90 vs. 78%, respectively. Clarithromycin resistance may reduce the efficacy of non-bismuth quadruple therapy, although the decrease in eradication rates seems to be far lower than in standard triple therapy (Gisbert and Cavet 2012).

#### Hybrid therapy

This therapy is based on 7 days of therapy with PPI and amoxicillin, followed by 7 days quadruple therapy with a PPI, amoxicillin, clarithromycin, and metronidazole. There are not many data in the literature, comparing this therapy with others, the standard or sequential therapies, but the results do not indicate that hybrid therapy will be superior to sequential therapy (Papastergiou et al. 2014a, b).

There is a review where five studies were identified comparing hybrid therapy and sequential therapy, and three comparing hybrid therapy and concomitant therapy. The five randomized controlled trials revealed no significant differences between hybrid therapy and sequential therapy, and the three randomized control trials showed no significant differences between hybrid therapy and concomitant therapy (He et al. 2015).

#### Levofloxacin-based therapies

Due to the increase of clarithromycin resistance, levofloxacin, a broad spectrum quinolone, is used for the *H. pylori* eradication in order to substitute clarithromycin in triple or sequential regimens. The eradication rate of therapies containing levofloxacin could be more than 90%, especially in areas where the local resistance to levofloxacin is low [less than 10%]. As for clarithromycin and metronidazole, an increase of levofloxacin resistance is being found, due to the fact that quinolones are often used for the treatment of urinary infections. The resistance to quinolones is around 20% in Europe, 15% in America, and 10% in Asia (Liang et al. 2014).

Due to the rapid development of secondary quinolone resistance, first-line use of levofloxacin is generally discouraged, and the drug is reserved for use in second-line regimens after failure of clarithromycin and/or metronidazole-based regimens (Gisbert et al. 2015).

#### Probiotic’s microorganism

Probiotics are defined as living microorganisms that, when administered in adequate amounts, can improve microbial balance in the intestine and exert positive health effects on the host (FAO/WHO 2002), including beneficial effects on the prevention of intestinal infections, cardiovascular disease, cancer, and anti-allergic effects (Pianoa et al. 2006).

Probiotics can be microorganisms from the bacteria or yeasts group. However, most of probiotics are bacteria, among them lactic acid bacteria, typically associated with the human gastrointestinal tract, which are the most widely used (Rodes et al. 2013b). They include Gram (+) cocci and rods *Lactobacillus* and *Bifidobacterium*, which are the two most common species used as probiotics and are extensively investigated for their beneficial effects on the host, including promotion of gut maturation and integrity, antagonism against pathogens, and modulation of the immune system and tumor-promoting agents. A common feature of these bacteria is the ability of anaerobic digestion of saccharides and production of lactic acid. These microorganisms are characterized by a resistance to low pH and tolerance to a wide range of temperatures. The natural ecosystem of the lactic acid bacteria is the digestive tract, mucous membrane of the mouth, and genital tract of humans and animals (Rodes et al. 2013a).

Due to fact that *H. pylori* has been regarded as a difficult-to-treat infection mainly because of acquired resistance to commonly used antibiotics, there is a growing interest in using probiotics in conjunction with antibiotic regimens to eradicate *H. pylori.* Probiotics have been proven to be useful in the treatment of several intestinal diseases such as diarrhea, in addition to the benefits of probiotic bacteria in the intestines; some beneficial effects on the stomach have been reported. Among them, the anti-*Helicobacter pylori* activity has been studied (Aiba et al. 2015).

The benefits of probiotic therapy in *H. pylori* case are increased eradication and improved tolerability by preventing the occurrence of treatment and related side effects.

Also, probiotics may help to improve the *H. pylori-*related diseases. The clinical outcome of *H. pylori* infection is determined by several factors, including the type of *H. pylori* strain, the extent of inflammation, and the density of *H. pylori* colonization. It has been reported that the risk of development of peptic ulcer disease and gastric cancer increases with an increasing level of infection. Therefore, permanent or long-term suppression of *H. pylori* could decrease the risk of developing *H. pylori-*related diseases (Yang et al. 2014).

Several studies have been conducted to show the favorable effect of different probiotics against *H. pylori* and have clarified the mechanism of action of probiotics against *H. pylori*, including strength of mucosal barrier, competition for adhesion, and immunomodulatory mechanisms (Papastergiou et al. 2014a, b).

#### Mechanism of action

##### Nonimmunological mechanism

The first line of defense against pathogenic bacteria is acidity of the stomach and the gastric mucosa barrier. It was suggested that, by taking probiotics, this first line of defense could be stronger due to the production of antimicrobial substances competing with *H. pylori* for adhesion receptors, stimulating mucin production and stabilizing the gut mucosal barrier.

##### Antimicrobial substances

Probiotics may inhibit *H. pylori* growth by secreting short chain fatty acids and antibacterial substances. Short chain fatty acids such as acetic, propionic, and lactic acids are produced during the carbohydrates metabolism by probiotics and as consequence, a pH reduction are found. In 1989, Bhatia et al. (1989) were the first group to observe an antagonistic effect of a *Lactobacillus* strain against *H. pylori* related to short chain fatty acids. Also, antimicrobial activity could be due to the inhibition of urease activity of *H. pylori* as has been shown in other publications (Sgouras et al. 2004).

Certain *Lactobacillus* species synthesize antimicrobial compounds related to the bacteriocin classes. Bacteriocins are proteinaceous toxins with potential anti-*H. pylori* activity. They are small and dialysable peptidic structures with antimicrobial activities. Antimicrobial activity of the bacteriocins varied among the *H. pylori* strains and also the type of bacteriocins produced by *Lactobacillus* sp. Some bacteriocins have shown a stronger antibacterial activity against *H. pylori* strains than others, although both were produced by *Lactobacillus* spp. (Kim et al. 2003).

##### Competition for adhesion

There are several possible mechanisms by which probiotic bacteria can inhibit the adhesion of *H. pylori.* The adhesion of *H. pylori* to epithelial cells is important in determining the outcome in *H. pylori*-associated diseases; the ability of the bacteria to establish physical contact with the gastric epithelium is affected by the influence of the epithelial mucosa, receptors associated with the adhesion of *H. pylori* to epithelium, and immune cells (Zhang et al. 2014).

There are several possible mechanisms by which probiotic bacteria can inhibit the adhesion of *H. pylori*, mainly lactic acid and bacteriocins (Lesbros-Pantoflickova et al. 2007).

##### Mucosal barrier

Mucosal surfaces have protective strategies to defend against noxious substances and pathogens found within the intestinal lumen. Some strategies, such as mucins, large complex glycoproteins that protect intestinal mucosal surfaces from microbial pathogens by limiting access of environmental matter to their epithelial cells (Mack et al. 2003). Several mucins have been identified. *H. pylori* is known to suppress MUCI and MUC5 gene expression in a human gastric cell line (Hanisch et al. 2014). It has been shown that in vitro studies with probiotics such as *L. plantarum* and *L. rhamnosus* increase the expression of MUC2 and MUC3 genes and therefore extracellular secretion of mucin by colon cell cultures can inhibit the adherence of pathogenic bacteria. This ability of these strains restores the mucosal permeability of gastric mucosa and inhibits the adherence of pathogenic bacteria such as *H. pylori*.

##### Immunologic mechanisms

The inflammatory response to gastric *H. pylori* infection is characterized by the release of various inflammatory mediators such as chemokines and cytokines. Probiotics could modify the immunologic response by the modulation of anti-inflammatory cytokines secretion, which would result in a reduction of gastric activity and inflammation (Wiese et al. 2012).

#### Clinical trials with probiotics against *H. pylori*

Reviewed papers related to probiotics and *H. pylori* have been search for in PUBMED for the last 10 years (from January 2004 to July 2017), by introducing keywords such as “review,” “probiotics,” “*Helicobacter*,” and “treatment”; 159 articles were obtained, but only 34 of them were related to probiotics and *H. pylori*. Based on the data found in these papers, probiotics cannot be considered as an alternative to anti-*H. pylori* treatment, but their use in association with standard anti-*H. pylori* treatment could improve the treatment of *H. pylori* by increasing eradication rates as well as decreasing the adverse effects of current medication therapy. This was the conclusion of the four meta-analyses related to *H. pylori* treatment and probiotics published between 2016 and 2017 (Lü et al. 2016, McFarland et al. 2016, Feng et al. 2017, Wang et al. 2017).

There is a scientific report published by Nature in 2016 that described the meta-analysis included eligible randomized controlled trials with the aim of determining whether probiotic supplementation can improve *H. pylori* eradication and reduce side effects during therapy (Dang et al. 2014). Authors investigated the effect of combining probiotics, with or without a placebo, without standard therapy. Compared to the placebo group, the probiotics group was eradication of *H. pylori* infection in intent-to-treat analysis and per protocol analysis, respectively*.* Probiotics with triple therapy plus a 14-day course of treatment did not improve the eradication of *H. pylori* infection compared to the placebo. However, probiotics did improve the adverse effects of diarrhea and nausea. These pooled dates suggest that the use of probiotics plus standard therapy does not improve the eradication rate of *H. pylori* infection compared to the placebo. Some meta-analysis has reported that probiotics supplementation can improve the eradication rate of *H. pylori* compared to the therapy alone. Maybe it is widely accepted that probiotics can improve *H. pylori* eradication and reduce side effects during standard therapy, but some species from a group of probiotic bacteria can help the pharmacotherapy. Consumption of fermented products in diet (f.ex. 250 g of milk fermented product) can improve gastrointestinal tract with good microorganism in the gastrointestinal tract. These data suggested that the use of probiotics plus standard therapy does not improve the eradication rate of *H. pylori* infection compared to the placebo. A placebo is a simulated or otherwise medically ineffectual treatment for a disease or other medical condition that intends to deceive the recipient. It is well known that physiological phenomena are closely associated with gastric diseases. In addition, the placebo effect generates alternation in the levels of systematic and enteric hormones and subject-expectancy effects (Dong et al. 2012; Lesbros-Pantoflickova et al. 2007; Ruggiero 2014; Vítor and Vale 2011; Patel et al. 2014; Sachdeva and Nagpal 2009; Zou et al. 2009; Franceschi et al. 2007; Lionetti et al. 2010; Zhengn et al. 2013; Patel et al. 2014; Pacifico et al. 2014; Sabbi 2011; Tong et al. 2007; Wilhelm et al. 2011; Gotteland et al. 2006). Meta-analysis shows that eradication rate of *H. pylori* infections in the probiotics group vs. the placebo and non-placebo groups in ITT analysis and estimates the OR with 95% covalence interval and weight percentage.

## Conclusion

Triple regimens containing two antimicrobial agents have been the standard therapies against *H. pylori* infection for more than 15 years. However, the increase in the prevalence of macrolide resistance, mainly clarithromycin, has decreased the efficacy of these therapies to unacceptably low levels in most parts of the world, resulting in the necessity of studying other possible therapies in order to eradicate the pathogen.

These therapies are the bismuth quadruple, sequential, and hybrid therapies. Due to the rapid development of quinolone resistance, levofloxacin-based regimens should be reserved as a second-line treatment option.

Related to probiotics, probiotics could not be recommended to be used as a single agent for eradication therapy. However, their use associated to standard treatment as an adjunct will improve the eradiation rates and decrease treatment-related side effects.
